# Artificial Selection of Early Emerging Helpers in the Cooperatively Breeding Ambrosia Beetle *Xyleborinus saxesenii* and Its Effects on Various Life History Traits and Their Fungal Symbionts

**DOI:** 10.1002/ece3.72356

**Published:** 2025-10-20

**Authors:** Antoine Melet, Peter H. W. Biedermann

**Affiliations:** ^1^ Chair of Forest Entomology and Protection, Faculty of Environment and Natural Resources Albert‐Ludwigs‐Universität Freiburg Germany

## Abstract

Overlapping generations is a defining characteristic of advanced social life. In cooperative breeding societies, temporary groups of mature offspring are formed that assist in the rearing of additional brood before dispersing and reproducing independently. It is hypothesized that the delayed dispersal period of helpers will determine the number of siblings that can be reared, thus resulting in an indirect fitness gain. The objective of this study was to investigate the effect of artificial selection for early dispersal of mature female helpers on the life history, behaviour and fungal symbionts in the ambrosia beetle Xyleborinus saxesenii. Two lineages were bred in the laboratory over the course of five generations, originating from the mixed offspring of two females caught in the wild. In one group, dispersing females were selected at random to initiate the next generation, while in the other group, only early dispersers were selected. A number of life‐history traits exhibited a pronounced response in the initial generation. Subsequently, these traits recovered to levels similar to those observed at the beginning of the experiment. Furthermore, significant differences in the fungal community were associated with our selection from the fourth generation onwards. The results suggest that X. saxesenii did not respond to our selection pressure. This may be due to the species' low genetic variability, which is the result of its sib‐mating habits, or its high phenotypic plasticity with regard to social behaviour.

## Graphical abstract

In cooperative breeding societies, temporary groups of mature offspring are formed that assist in the rearing of additional brood before the offspring disperse and reproduce independently. It is hypothesized that the delayed dispersal period of helpers will determine the number of siblings that can be reared, thus resulting in an indirect fitness gain. The objective of this study was to investigate the effect of artificial selection for early dispersal of mature offspring on the life history, behavior, and fungal symbionts in the ambrosia beetle *Xyleborinus saxesenii* .

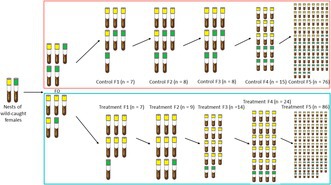

## 1 Introduction

A wide variety of animal species live in groups, ranging from the relatively simple herds of bison to the highly advanced hives of honeybees (Wilson 1971). Advanced sociality is defined by a number of features, including a high reproductive bias, the frequent occurrence of brood care by siblings (alloparental care), and a high degree of relatedness among helpers, reproducers, and offspring (Wilson 1971). The most derived social lifestyles are cooperative breeding and eusociality. In these societies, offspring typically do not disperse immediately after reaching maturity. Instead, they take over cooperative tasks in the natal nest, either permanently (eusociality) or for a limited period (cooperative breeding). Cooperatively breeding species combine philopatry, where mature offspring stay in the nest before dispersing, with alloparental care. Therefore, it is not surprising that alloparental care has evolved alongside philopatry in such societies (Hochberg et al. 2008; Le Galliard et al. 2005; Mullon et al. 2018). This theoretical assumption is also supported by correlative evidence in a wide range of social organisms ranging from microbes to vertebrates (Choe and Crespi 1997; Fisher et al. 2013; Hamilton 1964; Hatchwell 2009; Koenig and Dickinson 2016; Rainey and Rainey 2003; Taborsky 1994). Despite this, there is currently little experimental evidence for the co‐evolution of philopatry and alloparental care.

*Xyleborinus saxesenii* Ratzeburg, 1837 (Curculionidae: Scolytinae: Xyleborini) is a Eurasian ambrosia beetle that lives in cooperatively breeding societies. These societies exhibit variable durations of female offspring philopatry and alloparental care (Biedermann and Taborsky 2011). Adult female offspring typically delay dispersal after fertilization by their brothers. While staying in their natal nest, daughters assist their mother with brood care, fungus cultivation, and tunnel enlargement (Biedermann and Taborsky 2011; Peer and Taborsky 2007). The daughters' dispersal decisions differ, staying in their natal nest for 17 to 38 days depending on the presence of brood (Biedermann, Klepzig, and Taborsky 2011). However, the reasons why some females disperse earlier than others are unknown. In *Xyleborus affinis* Eichhoff, a related species with a similar social lifestyle, a long philopatric period has been shown to be associated with a direct fitness cost later in life (Biedermann, Klepzig, and Taborsky 2011). Both species can be reared in an artificial medium (Biedermann et al. 2009), providing a unique opportunity to select for short philopatric periods in adult offspring and thus test for correlated effects on alloparental care. Ambrosia beetles behave socially under these conditions, but artificial rearing conditions are certainly more favorable than natural wood in terms of the softness and nutritional value of the tissues and the higher, more stable temperatures (Biedermann et al. 2009). This may result in less cooperation when boring tunnels and tending to nutritional fungi than would occur under natural conditions. However, it is not possible to select ambrosia beetles inside wood experimentally.

Ambrosia beetles are typically associated with mutualistic fungi, which they cooperatively cultivate in monocultures within tunnel systems and which serve as food for both larvae and adults (Biedermann and Vega 2020). *Xyleborinus saxesenii* cultivates two food fungi, *Dryadomyces sulphureus* and *Raffaelea canadensis* (Biedermann et al. 2013; Diehl et al. 2022; Francke‐Grosmann 1975), which develop in succession. Other fungal species are also present in *X. saxesenii* nests, although they are not as closely associated with the beetle (Biedermann et al. 2013). However, the accumulation of less beneficial fungi within nests over time also affects the fitness of late‐dispersing offspring, who vertically transmit all these fungi to newly established nests (Diehl et al. 2022). A strong correlation has been found between highly productive nests, their durability, long philopatric periods, and alloparental care by adult daughters (Biedermann and Taborsky 2011; Nuotclà et al. 2021). Therefore, it has been hypothesized that the management of beneficial fungal communities is an important factor for nest durability and consequently for the philopatry in ambrosia beetles (Biedermann and Rohlfs 2017).

Artificial selection experiments are a valuable research tool for studying evolutionary processes. Phenotypic responses can be measured as a direct response to selective pressure (Brakefield 2003; Conner 2003). Under natural conditions, adaptation is typically too slow to be observed within the time frame of a scientific project or even a lifetime. By applying strong selection pressure under controlled conditions, researchers can detect adaptive change within a few generations (Conner 2003). Combined with the relatively short generation time of certain insects, it is possible to observe adaptation over a period of just a few months. The use of artificial selection experiments allows for direct experimental testing of hypotheses that can be used to complement the results of correlative studies (Lewis and Morran 2022). Such experiments are also used to study communities of two or more species (Blouin et al. 2015; Swenson et al. 2000).

The aim of this study was to examine the relationship between short philopatric periods in adult female offspring and the expression of other life history traits in the subsequent generation of offspring, with a particular focus on alloparental care and nest founding success. Pilot results showed that early dispersal in *X. saxesenii* was heritable and associated with differences in social behavior (Taborsky et al. 2021). We hypothesized that beetles selected for early dispersal would show a tendency to disperse earlier and earlier over generations and would have nests that collapse sooner, produce fewer offspring, and show a reduction in alloparental care. We also sought to determine the effects of offspring philopatry on fungal communities. It was hypothesized that these communities would be less beneficial in nests of females with long philopatric periods and that they would contain higher relative abundances of *R. canadensis* than *D. sulphureus* fungal cultivars (Diehl et al. 2022).

## 2 Material and Methods

### 2.1 Model Species

*Xyleborinus saxesenii* females emerge from their native nest already mated and proceed to initiate new nests by excavating into suitable pieces of wood. They tunnel deep into the xylem and inoculate the wood with mutualistic fungi that they have transported from their native nest in mycetangia (specialized organs for transporting fungal spores) and guts (Batra 1966; Francke‐Grosmann 1975; Mayers et al. 2022). Once the fungi are established, the foundress begins to lay eggs. The mutualistic fungi serve as the food source for the adults and to a large extent also for the larvae (i.e., larvae feed on fungus‐infested xylem; De Fine Licht and Biedermann 2012). After pupation, adults mate with their siblings. Afterwards, they exhibit a philopatric period during which they show cooperative behaviors before dispersing (Biedermann, Peer, and Taborsky 2011). Males only rarely leave the nest to mate with unrelated individuals, so inbreeding is likely to be very high. This is a common phenomenon in Xyleborinii and is tolerated by the species, as deleterious mutations are fully expressed in the haploid males and thus eliminated from the population (Biedermann 2010).

Most nests last for 3 months from initiation until the last offspring disperses, though some last several months (Biedermann, Klepzig, and Taborsky 2011). Due to this generation length and the need for an overwintering diapause (see below), only five generations could be bred during the 2 years this study spanned.

### 2.2 Artificial Rearing

Beetles were captured in a forest near Würzbürg, Germany, and brought to the lab for rearing in artificial medium. All captured females were briefly immersed in 70% ethanol and rinsed in distilled water to remove the ethanol. The primary fungal mutualists are not adversely affected by this sterilization protocol (Biedermann et al. 2009) as they are protected within the mycetangia. The beetles were then briefly dried on sterile tissue paper and immediately transferred individually to rearing tubes. The resulting nests were designated as “nests of wild‐caught females.”

The rearing tubes were transparent plastic tubes, filled to two‐thirds capacity with standard artificial rearing media composed of beech sawdust, agar, and additional nutrients (Biedermann et al. 2009). The tubes were sealed with “drosophila plugs”, which are gas‐permeable sponge stoppers made of high‐density foam.

All nests were individually labeled with unique identifiers, and their date of founding was recorded. Nests were observed three times per week from the time of foundation until all beetles had emerged. All nests in this experiment were maintained at a constant temperature of 25°C, 70% humidity, and darkness. Mature and sib‐mated females left the nest after a variable philopatric period. Because the tubes were closed, females attempting to disperse could not exit the tube and were thus trapped between the artificial medium and the plug. We collected these females every second day, surface sterilized them as described above, and put them individually in fresh rearing tubes to initiate a new generation.

Pilot studies indicated that periods of diapause are essential for the successful long‐term rearing of *X. saxesenii* . Therefore, during the second generation (F2), the breeding temperature was lowered to 8°C for 10 weeks to simulate the overwintering period. Egg‐laying ceased during this period. Only larvae and adults were observed in the diapausing galleries, along with dead pupae that were rapidly cannibalized at the end of diapause. No dispersal was observed during diapause.

### 2.3 Directional Artificial Selection

**FIGURE 1:**
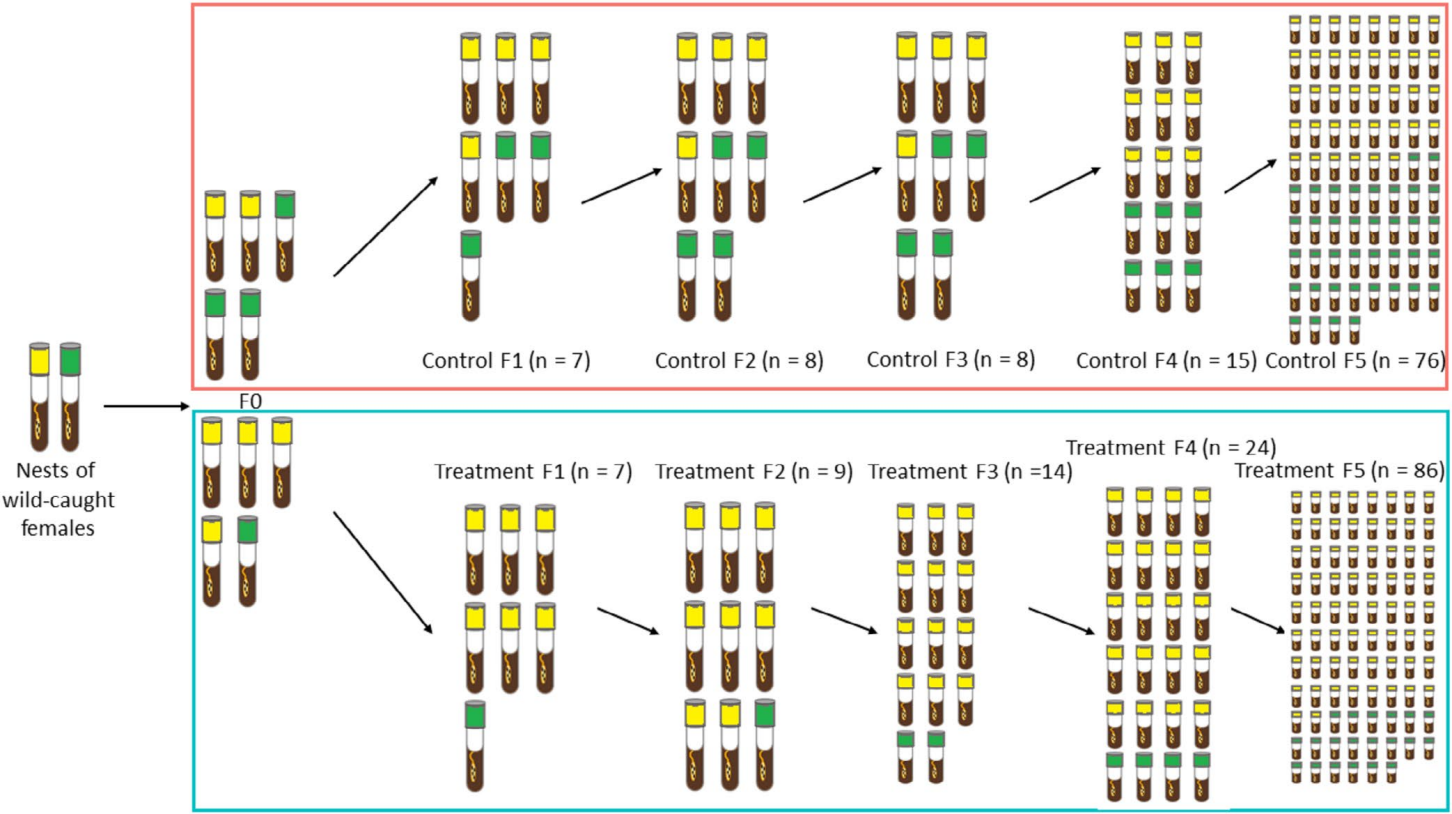
Directional artificial selection. Sample sizes show the number of nests used for the life history and behavioral analysis. In nests assigned to the treatment lineage (blue frame), we always selected the first seven dispersing females as foundresses for the next generation, while in the control lineage (pink frame), we randomly selected seven dispersing females. Nests showed with yellow lids descend from W5, nests showed with green lids descend from W13.

All daughter females that dispersed from two initial nests of wild‐caught females were collected and used to initiate the F0 generation of the experiment. To favor an extreme phenotype of interest (early dispersal of females), two lineages were created. The treatment lineage was subjected to artificial selection, while the control lineage was not. The control lineage represented a moderate phenotype for comparison with the extreme phenotype from the treatment lineage. This selection protocol resulted in directional artificial selection. From F0, 5 nests were randomly assigned to the treatment lineage and 5 nests were randomly assigned to the control lineage (Figure 1).

In nests assigned to the treatment lineage, we always selected the first seven females to disperse as foundresses for the next generation, while in the control lineage we randomly selected seven dispersing females. This process was consistently repeated for five generations, resulting in the groups “treatment F1” to “treatment F5” and “control F1” to “control F5” (Figure 1). Using this method, up to 15% of the nests produced a new generation of beetles that developed and reached the adult stage. These beetles then dispersed and contributed to the next generation. Nest failure usually occurs within the first few days after initiation, as the mutualistic fungi fail to establish. Nests that failed to produce dispersing females were excluded from our analysis. This resulted in the following sample sizes for the different groups: control F1 (*N* = 7), F2 (*N* = 8), F3 (*N* = 8), F4 (*N* = 15), F5 (*N* = 76), and treatment F1 (*N* = 7), F2 (*N* = 9), F3 (*N* = 14), F4 (*N* = 23), F5 (*N* = 86). We used these sample sizes for all life history and behavioral analyses.

For each female that initiated a new nest, the nest of origin was recorded, allowing us to establish the ‘genealogy’ of each nest. All the nests were traced back to two nests of wild‐caught females (W5 and W13, see Figure 1, Tables S1 and S2). This lineage origin was included as a random factor in subsequent analyses to account for possible effects due to the nest of origin. In our analyses, we examined (i) the effect of the selection at each step, by comparing successive generations within each lineage (control F1 vs. F2, F2 vs. F3, F3 vs. F4, F4 vs. F5 and treatment F1 vs. F2, F2 vs. F3, F3 vs. F4, F4 vs. F5), (ii) the ultimate effect of the selection, by comparing the first and last generations within each lineage (control F1 vs. F5 and treatment F1 vs. F5), and (iii) the divergence between the two lineages, by comparing the two groups within each generation (control F1 vs. treatment F1, control F2 vs. treatment F2, control F3 vs. treatment F3, control F4 vs. treatment F4, control F5 vs. treatment F5). Special attention was paid to the first generation in order to identify changes that occur early in the selection process.

### 2.4 Nest Development

The number of eggs, larvae, pupae, adult females in the nest and dispersing females was recorded for each observation. The three larval instars were not distinguished. A nest was considered to have reached the end of its lifespan when no adults or dispersers were observed for seven consecutive days. Last observations that did not include a record of adults or dispersers were excluded from the dataset to avoid artificially inflating the lifespan of nests. Two key variables were extracted from the census data: (i) total nest lifespan, defined as the number of days between the start day and the day the last disperser was observed, and (ii) timing of first dispersal, defined as the number of days between the start day and the day the first disperser was observed.

Developmental times were compared using multi‐level generalized linear models with Poisson distribution. To examine the effects of the directional artificial selection (“control” or “treatment”) on the nest development, the total lifespan and the timing to first dispersal were set as the response variables, respectively. The directional artificial selection was set as an explanatory variable. We included the nests lineage (“W5” or “W13”) as a random variable to account for possible lineage effects. The *p*‐values resulting from these models were corrected for multiple comparisons using Tukey contrasts.

Dispersal patterns were compared using pairwise survival analysis, which was based on a Cox proportional hazards model. To examine the effects of the directional artificial selection (“control” or “treatment”) on the dispersal patterns, the dispersal of individual females was set as the response variable. The directional artificial selection was set as an explanatory variable. We included the nests lineage (“W5” or “W13”) as a random variable to account for possible lineage effects. As several females disperse from each nest, we included the nest ID as a random variable.

### 2.5 Productivity

The total number of dispersers collected from a nest was used as the productivity of that nest. Productivities were compared using a multi‐level generalized linear model with Poisson distribution. To examine the effects of the directional artificial selection (“control” or “treatment”) on the productivity, the productivity was set as the response variable. The directional artificial selection was set as an explanatory variable. We included the nests' lineage (“W5” or “W13”) as a random variable to account for possible lineage effects. The *p*‐values resulting from these models were corrected for multiple comparisons using Tukey contrasts. The effect of the timing of first dispersal on the productivity was analyzed using a linear model, using the productivity of the nest as the response variable and the time of first dispersal as the explanatory variable.

### 2.6 Behavioral Observations

After each count of a nest, a scan observation was made to record the behavior of each larva and adult encountered, following the method described by Biedermann and Taborsky (2011). The behavior of larvae and adults was recorded separately. The duration of the scan observations was kept to a minimum in order to obtain a snapshot of the behavior of the entire nest. No behavioral data were recorded between the dates of 11.07.2022 and 18.07.2022, and between 08.08.2022 and 15.08.2022. From the behavioral recordings, two categories of behaviors were analyzed: (i) activity, defined as all the behaviors except resting behavior, and (ii) social behavior, defined as the behaviors directed towards nest excavation and hygiene (i.e., digging, balling, grooming, and cannibalism for the larvae and digging, shuffling, grooming, and cannibalism for the adults).

Activity and social behavior of larvae and adult females were analyzed separately using multi‐level generalized linear models with binomial error distributions. To examine the effects of the directional artificial selection (“control” or “treatment”) on the behavior, the larval and adult social behaviors and the larval and adult activity levels were set as the response variables, respectively. The directional artificial selection was set as the explanatory variable. We included the nest lineage (“W5” or “W13”) as a random variable to account for possible lineage effects. The *p*‐values resulting from these models were corrected for multiple comparisons using Tukey contrasts.

### 2.7 Fungal Community Sampling and DNA Extraction

Beginning with the second generation, nests were randomly selected from each group. The first seven dispersing females from the selected nests were collected because the males lack mycetangia and do not transport fungal spores (Francke‐Grosmann 1975). We pooled seven females to make sure we collected enough fungal material. The mycetangia of a single beetle are small and may not contain enough material for proper detection (Batra 1963). Pooling females also helped reduce artifacts due to within‐nest variability. After collection, dispersing females were frozen at −20°C until DNA extraction and fungal community sequencing. Some nests did not produce dispersers, resulting in the following final sample sizes for the different groups: control F2 (*N* = 7), F3 (*N* = 8), F4 (*N* = 8), F5 (*N* = 8), and treatment F2 (*N* = 7), F3 (*N* = 6), F4 (*N* = 7), F5 (*N* = 7). The first generation was not included due to a lack of available nests. Nests selected for the fungal community analysis were excluded from the life history and behavioral analyses.

To extract fungal DNA, the seven females that dispersed from a nest were pooled into a single sample. The samples were subjected to mechanical grinding at 2700 rpm for 20 min in a ZR BashingBead Lysis Tube 2.0 mm with 750 μL of lysis solution (Zymo Research, Germany) on a Vortex Genie 2 (Scientific Industries). After centrifugation at 18,000 g for 1 min, the supernatant was collected and transferred to a ZR BashingBead Lysis Tube 0.1 and 0.5 mm. Thereafter, 300 μL of lysis solution was added, and the mixture was vortexed at 2700 rpm for 20 min on a Vortex Genie 2. DNA was then extracted using ZymoBIOMICS DNA Miniprep kits (Zymo Research, Germany) according to the manufacturer's instructions. The isolated DNA was stored at −20°C until further processing.

### 2.8 Amplicon Sequencing of Fungal Communities

The amplification of fungal DNA was achieved by using LSU (28S) rRNA primers, since the primers typically used for the analysis of fungal communities, targeting the ITS region, proved ineffective for the amplification of Ophiostomataceae species, including *Raffaelea* and *Dryadomyces* (Kostovcik et al. 2015). The dual‐index primers of LIC15R and nu‐LSU‐355‐30 are described in the electronic supplementary material of the first study in which they were used (Nuotclà et al. 2021). The PCR conditions were as follows: an initial denaturation at 98°C for 3 min, 35 cycles of denaturation at 98°C for 10 s, annealing at 54°C for 30 s, and elongation at 72°C for 20 s; followed by a final extension at 72°C for 10 min.

After amplification, DNA samples were sequenced by StarSeq (Mainz, Germany) using the described primers on the Illumina MiSeq v3 2 × 300‐bp platform according to the Illumina protocol. To ensure the quality of our fungal community results, we included negative and positive controls along with our samples. The negative controls were prepared and sterilized as previously described but lacked the presence of the beetles. Two negative controls were prepared alongside the samples from the control F5 and treatment F5 groups. In addition, the sequencing company utilized two samples of pure water, which were devoid of any DNA and which underwent all library preparation steps. The positive control was a mock community that had been prepared for another experiment and sent for sequencing within the same batch. The mock community consisted of equal amounts of *Dryadomyces sulphureus, Raffaelea canadensis, Beauveria bassiana*, and the yeasts *Pichia* sp. and *Candida* sp. DNA was extracted as previously described. The positive control provided by the company consisted of a defined amount of DNA from eight bacterial species (*Pseudomonas aeruginosa, Escherichia coli, Salmonella enterica, Lactobacillus fermentum, Enterococcus faecalis, Staphylococcus aureus, Listeria monocytogenes, Bacillus subtilis*) and two yeast species (*Saccharomyces cerevisiae, Cryptococcus neoformans*) (ZymoBIOMICS Microbial community DNA standard, Zymo Research, Germany). The mock community and the positive control provided by the company were replicated twice, resulting in a total of four positive controls.

### 2.9 Bioinformatics Processing

The raw, demultiplexed reads were processed using Usearch v11 (Edgar 2010). The forward and reverse reads were merged using the *‐fastq_mergepairs* command, with a minimum of 200 base pairs for the merged sequence and a maximum of 20 mismatches in the alignment as a preliminary quality filtering step. The primers were truncated from the reads using the *‐fastx_truncate* command, and the overall quality was then assessed using the *‐fastq_filter* command, with an expected total error threshold of 1. Unique sequences were identified using the *‐fastx_uniques* command, and the singletons were excluded using the ‐*sortbysize* command with a minimum size of 2. The amplicon reads were denoised using the ‐*unoise3* command. This command does not cluster similar sequences; rather, it identifies and corrects reads with sequencing errors and removes chimeras, resulting in amplicon sequence variants (ASVs), a higher resolution analog of the traditional OTU (Edgar 2016b). The ASVs were taxonomically classified in two steps using the *‐usearch_global* command. The command is based on the USEARCH algorithm, which searches for high identity hits to a database sequence (Edgar 2010). In the first step, a database of LSU sequences from stock cultures of fungi isolated from *X. saxesenii* was used. The database contained 18 reference sequences from 12 fungal species (Diehl et al. 2022). The identity threshold was set at 97% due to the fact that the database includes sequences of known fungal symbionts of *X. saxesenii* . The unclassified ASVs were used as the input for the second step, which used a custom reference database constructed from NCBI data using BCdatabaser (Keller et al. 2020). The second database contained 82,250 sequences (Diehl et al. 2022). To ensure accurate classification, the identity threshold was set at 99%. In cases where the taxonomic outputs differed between the two steps, the output from the first step was retained. The remaining unclassified ASVs were then used as input for the final step, using the *‐sintax* command with a sintax cutoff of 0.8. The SINTAX algorithm is comparable to a naive Bayesian classifier algorithm, but it does not require training (Edgar 2016a).

### 2.10 Statistical Analysis of Metabarcode Data

To improve the quality of our dataset, we applied a contaminant removal method according to the R package ‘decontam’, taking into account the negative controls. This process reduces the complexity of the microbiome data in downstream analyses while preserving its integrity (Davis et al. 2018). The positive and negative controls were visualized but excluded from the sample set. After decontamination and the exclusion of the controls, the samples were rarefied, accounting for unequal numbers of reads between samples. The sample with the fewest reads had 9838 reads; this threshold was used to generate rarefied samples for subsequent analysis.

The alpha diversity of the fungal communities was compared between the different groups by comparing the observed ASV richness and Shannon's diversity index using multilevel generalized linear models, followed by a multiple comparison using Tukey contrasts. The beta diversity was analyzed by calculating dissimilarity matrices using the Bray‐Curtis method, taking into account both the presence/absence and relative abundances of ASVs. A pairwise PERMANOVA was used to analyze the matrices. Composition barplots were constructed for visualization, aggregated to the genus level and faceted by group.

### 2.11 Statistical Softwares

All statistical tests were performed using R version 4.0.2 (R Core Team 2020), with the RStudio interface version 1.3.1073. The additional packages *car* (Fox and Weisberg 2019), *decontam* (Davis et al. 2018), *lme4* (Bates et al. 2015), *multcomp* (Hothorn et al. 2008), *pairwiseAdonis* (Martinez Arbizu 2020), *phyloseq* (McMurdie and Holmes 2013), *rstatix* (Kassambara 2023), *survival* (Therneau and Grambsch 2000) and *vegan* (Oksanen et al. 2022) were used.

## 3 Results

Although the goal of directional artificial selection was to accelerate female dispersal over five generations, no significant difference in dispersal time was observed between the two lineages (control F5 vs. treatment F5, GLM, *z* = 0.842, *p* = 1).

### 3.1 Lifespan of Nests

**FIGURE 2:**
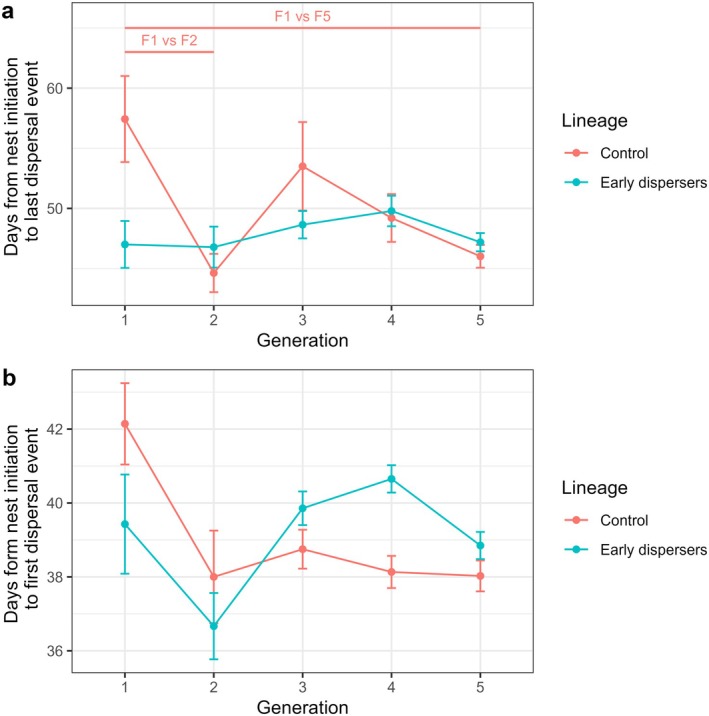
Development of the two lineages, from F1 to F5. The lifespan (a) is expressed as the average number of days between the foundation of the nest and the dispersal of the last offspring. The delay to dispersers (b) is the average number of days between the foundation of the nest and the observation of the first dispersing offspring. The vertical bars show the standard errors of the mean. The number of nests used for each group is as follows: Control F1 (*N* = 7), F2 (*N* = 8), F3 (*N* = 8), F4 (*N* = 15), F5 (*N* = 76); Treatment F1 (*N* = 7), F2 (*N* = 9), F3 (*N* = 14), F4 (*N* = 23), F5 (*N* = 86). Annotations on top of the plot indicate the groups that differ significantly. Groups that differ in the control lineage are shown in pink.

In the control lineage, nest lifespan was 57.43 days in F1, longer than in F5 (control F1 vs. F5, GLM, *z* = −4.21, *p* < 0.001), and longer than in F2 (control F1 vs. F2, GLM, *z* = −3.47, *p* = 0.02). In the control lineage, no other two successive generations were found to be significantly different (GLMs, all *p* > 0.05). In the treatment lineage, nest lifespan was 47 days in F1, not significantly shorter than in F5 (treatment F1 vs. F5, GLM, *z* = 0.07, *p* = 1), and no two successive generations were found to be significantly different (GLMs, all *p* > 0.05). The two lineages were not significantly different in any of the generations (GLMs, all *p* > 0.05) (Figure 2, Table S3).

### 3.2 Time Before the Start of Dispersal

In the control lineage, the duration before the start of dispersal was 42.14 days in F1, not significantly longer than in F5 (GLM, *z* = −1.68, *p* = 0.78). In the control lineage, no two successive generations were found to be significantly different (GLMs, all *p* > 0.05). In the treatment lineage, the duration before the start of dispersal was 39.43 days in F1, not significantly longer than in F5 (GLM, *z* = −0.24, *p* = 1), and no two successive generations were found to be significantly different (GLMs, all *p* > 0.05). The two lineages were not significantly different in any of the generations (GLMs, all *p* > 0.05) (Figure 2, Table S4).

### 3.3 Timing of Dispersal

**FIGURE 3:**
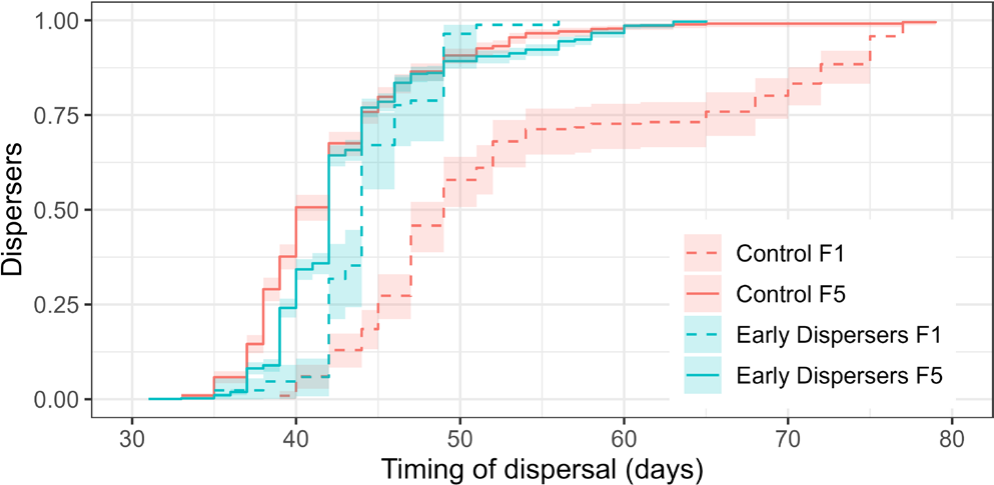
Dispersal pattern of the two lineages in generations F1 and F5. The y‐axis shows the proportion of the total dispersers that left their native nest. The timing of dispersal is expressed as the number of days from the foundation of the nest. Colored areas indicate the confidence intervals. The number of nests used for each group is as follows: Control F1 (*N* = 7), F2 (*N* = 8), F3 (*N* = 8), F4 (*N* = 15), F5 (*N* = 76); Treatment F1 (*N* = 7), F2 (*N* = 9), F3 (*N* = 14), F4 (*N* = 23), F5 (*N* = 86).

In the control lineage, dispersal occurred later in F1 than in F5 (control F1 vs. F5, Cox model, *z* = 4.264, *p* < 0.001) and in F2 (control F1 vs. F2, Cox model, *z* = 3.558, *p* = 0.012); no other two successive generations were found to be significantly different (Cox model, all *p* > 0.05). In the treatment lineage, no two successive generations were found to be significantly different (Cox models, all *p* > 0.05). A comparison of the two lineages showed that dispersal occurred later in control F1 than in treatment F1 (control F1 vs. treatment F1, Cox model, *z* = 3.577, *p* = 0.012). The two lineages were not significantly different in any of the other generations (Cox models, all *p* > 0.05) (Figure 3).

### 3.4 Productivity

**FIGURE 4:**
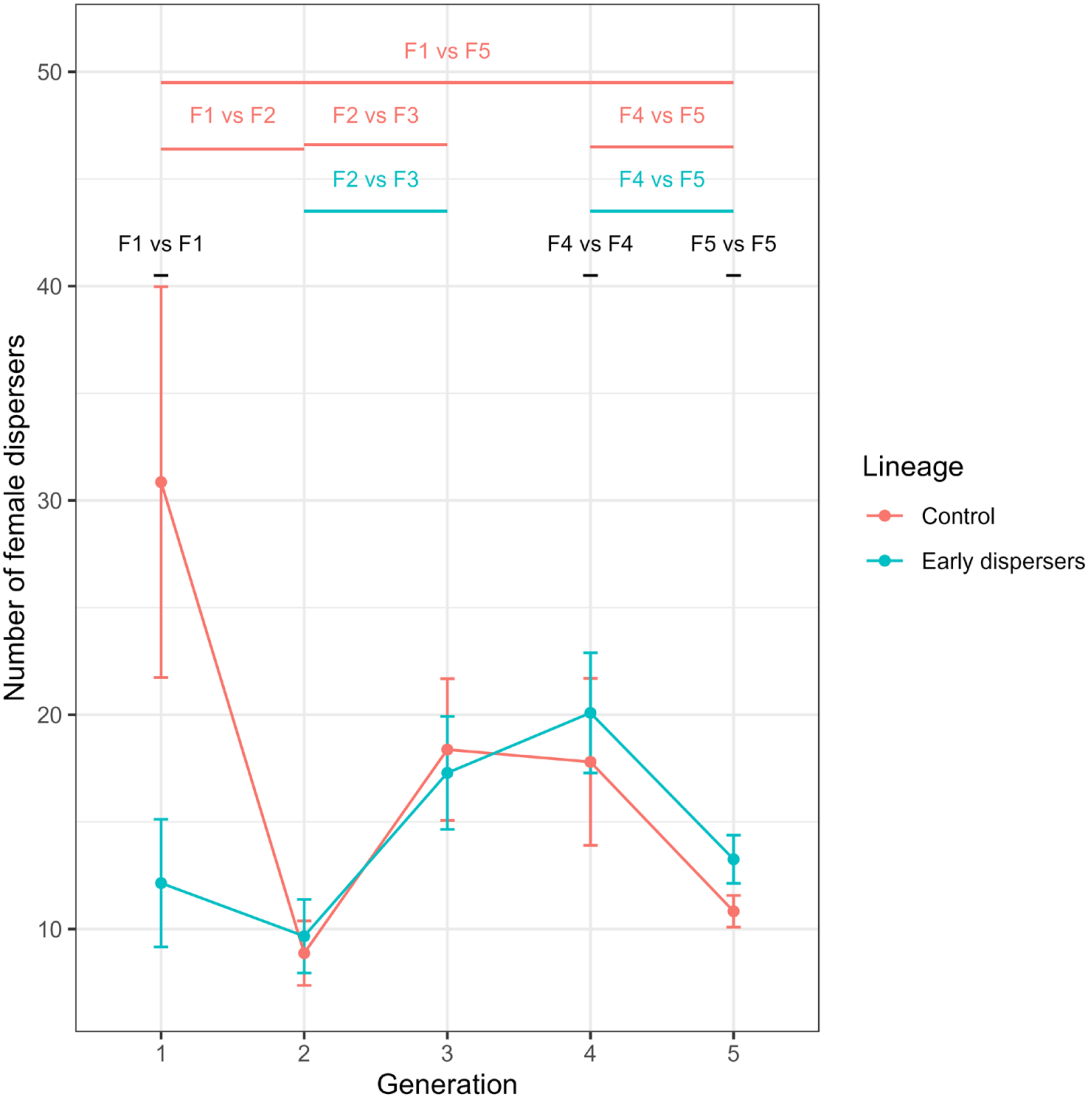
Productivity of the two lineages, from F1 to F5. The productivity is expressed as the average number of females that dispersed from the nests. The vertical bars show the standard errors of the mean. The number of nests used for each group is as follows: Control F1 (*N* = 7), F2 (*N* = 8), F3 (*N* = 8), F4 (*N* = 15), F5 (*N* = 76); Treatment F1 (*N* = 7), F2 (*N* = 9), F3 (*N* = 14), F4 (*N* = 23), F5 (*N* = 86). Annotations on top of the plot indicate the groups that differ significantly. Groups that differ are shown in pink and blue for differences in the control and treatment lineages respectively. If the lineages differ for a given generation, the annotation is black.

In the control lineage, productivity was 30.86 dispersing females per nest in F1, higher than in F5 (GLM, *z* = −15.27, *p* ≤ 0.001), and higher than in F2 (GLM, *z* = −9.38, *p* < 0.001). Productivity was lower in F2 than in F3 (GLM, *z* = 5.04, *p* < 0.001), and higher in F4 than in F5 (GLM, *z* = −9, *p* < 0.001). In the control lineage, productivity was not significantly different between F3 and F4 (GLM, *p* = 1). In the treatment lineage, productivity was 12.14 dispersing females per nest in F1, not significantly lower than in F5 (GLM, *z* = 0.29, *p* = 1). Productivity was lower in F2 than in F3 (GLM, *z* = 4.49, *p* < 0.001) and higher in F4 than in F5 (GLM, *z* = −8.17, *p* < 0.001), but no other two successive generations were found to be significantly different (GLMs, all *p* > 0.05). Productivity was higher in control F1 than in treatment F1 (GLM, *z* = −5.97, *p* < 0.001), lower in control F4 than in treatment F4 (GLM, *z* = 3.24, *p* = 0.034), and lower in control F5 than in treatment F5 (GLM, *z* = 9.03, *p* < 0.001). The two lineages were not significantly different in the other generations (GLMs, all *p* > 0.05) (Figure 4, Table S5).

Overall, productivity was strongly correlated with the timing of dispersal (linear model, *z* = 5.55, *p* < 0.001).

### 3.5 Behavioral Observations

**FIGURE 5:**
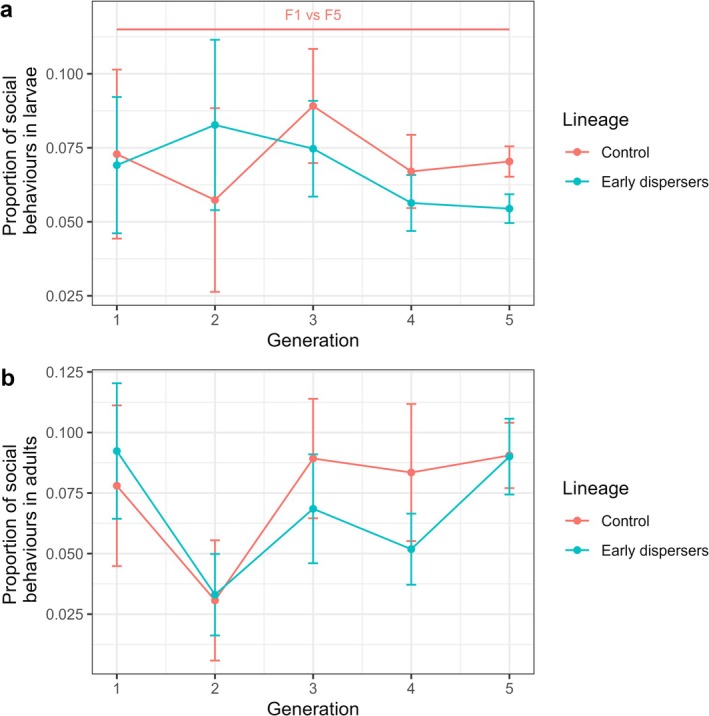
Social behaviors of the two lineages, from F1 to F5 in (a) larvae and (b) adults. The proportion of social behaviors is expressed as the number of behaviors directed to nest hygiene and allogrooming, out of all behaviors recorded. The vertical bars show the standard errors of the mean. The number of nests used for each group is as follows: Control F1 (*N* = 7), F2 (*N* = 8), F3 (*N* = 8), F4 (*N* = 15), F5 (*N* = 76); Treatment F1 (*N* = 7), F2 (*N* = 9), F3 (*N* = 14), F4 (*N* = 23), F5 (*N* = 86). Annotations on top of the plot indicate the groups that differ significantly. Groups that differ in the control lineage are shown in pink.

In the control lineage, the proportion of larval social behaviors was higher in F1 than in F5 (GLM, *z* = −3.42, *p* = 0.02). In the control lineage, no two successive generations were found to be significantly different (GLMs, all *p* > 0.05). In the treatment lineage, the proportion of larval social behaviors was not significantly higher in F1 than in F5 (GLM, *z* = −1.49, *p* = 0.87). In the treatment lineage, no two successive generations were found to be significantly different (GLMs, all *p* > 0.05). The two lineages were not significantly different in any of the generations (GLMs, all *p* > 0.05) (Figure 5A, Table S6).

Similarly, in the control lineage, the proportion of adult social behaviors was not significantly lower in F1 than in F5 (GLM, *z* = −2.45, *p* = 0.26). In the control lineage, no two successive generations were found to be significantly different (GLMs, all *p* > 0.05). In the treatment lineage, the proportion of adult social behaviors was not significantly higher in F1 than in F5 (GLM, *z* = −0.21, *p* = 1). In the treatment lineage, no two successive generations were found to be significantly different (GLMs, all *p* > 0.05). The two lineages were not significantly different in any of the generations (GLMs, all *p* > 0.05) (Figure 5B, Table S7).

**FIGURE 6:**
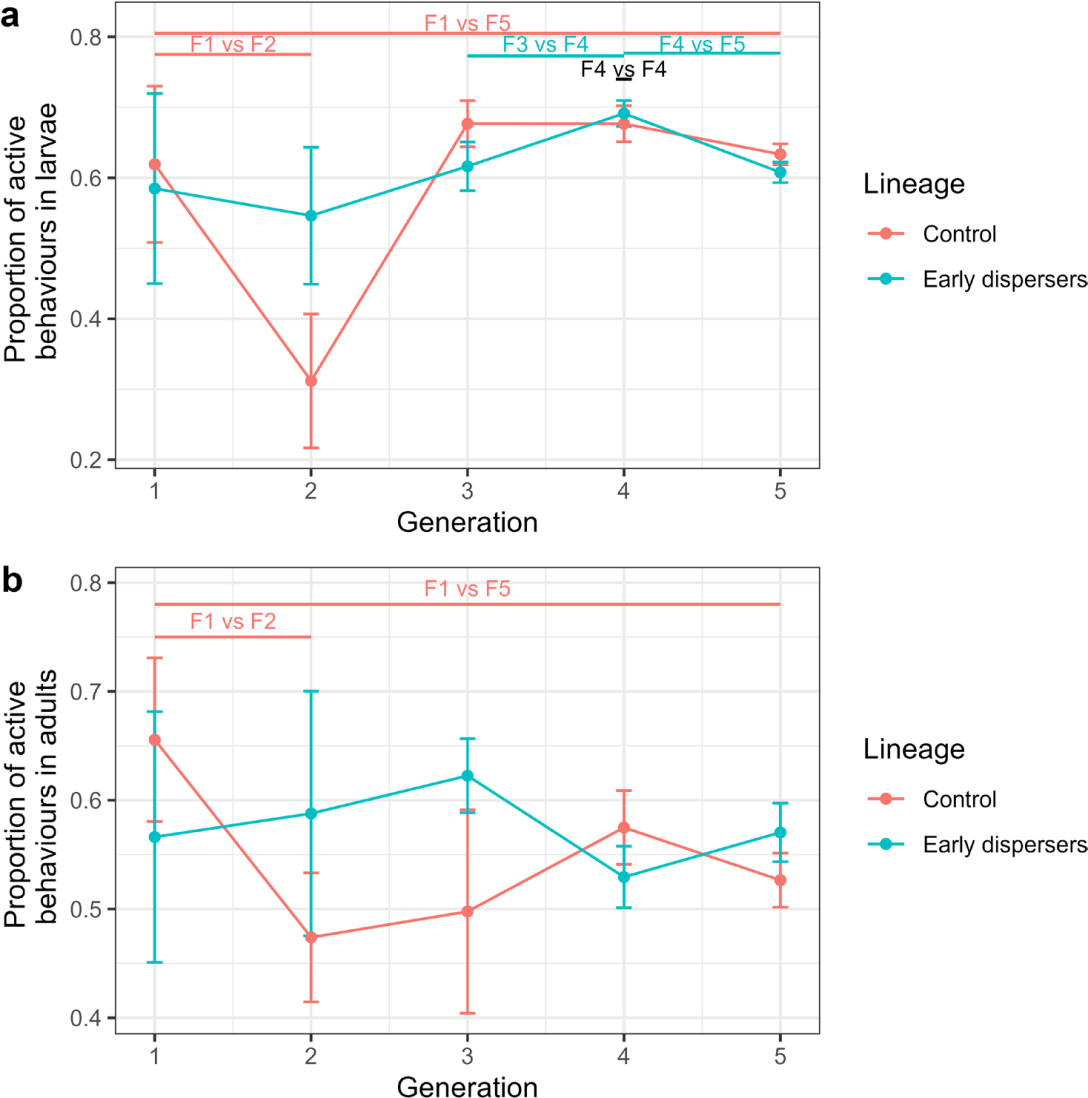
Active behaviors of the two lineages, from F1 to F5 in (a) larvae and (b) adults. The proportion of active behaviors is expressed as the number of behaviors recorded, excluding the resting behavior. The vertical bars show the standard errors of the mean. The number of nests used for each group is as follows: Control F1 (*N* = 7), F2 (*N* = 8), F3 (*N* = 8), F4 (*N* = 15), F5 (*N* = 76); Treatment F1 (*N* = 7), F2 (*N* = 9), F3 (*N* = 14), F4 (*N* = 23), F5 (*N* = 86). Annotations on top of the plot indicate the groups that differ significantly. Groups that differ are shown in pink and blue for differences in the control and treatment lineages respectively. If the lineages differ for a given generation, the annotation is black.

In the control lineage, the proportion of larval active behaviors was higher in F1 than in F5 (GLM, *z* = 4.74, *p* < 0.001), and higher in F1 than in F2 (GLM, *z* = 4.24, *p* < 0.001). In the control lineage, no other two successive generations were found to be significantly different (GLMs, all *p* > 0.05). In the treatment lineage, the proportion of larval active behaviors was not higher in F1 than in F5 (GLM, *z* = 2.66, *p* = 0.16). In the treatment lineage, the proportion of larval active behaviors was lower in F3 than in F4 (GLM, *z* = −3.12, *p* = 0.045), and higher in F4 than in F5 (GLM, *z* = 5.75, *p* < 0.001), but no two other successive generations were found to be significantly different (GLMs, all *p* > 0.05). The proportion of larval active behaviors was lower in control F4 than in treatment F4 (GLM, *z* = −3.53, *p* = 0.012), and the two lineages were not significantly different in any of the other generations (GLMs, all *p* > 0.05) (Figure 6A, Table S8).

In the control lineage, the proportion of adult active behaviors was higher in F1 than in F5 (GLM, *z* = 3.95, *p* < 0.001), and higher in F1 than in F2 (GLM, *z* = 3.8, *p* < 0.001). In the control lineage, no other two successive generations were found to be significantly different (GLMs, all *p* > 0.05). In the treatment lineage, the proportion of adult active behaviors was not significantly lower in F1 than in F5 (GLM, *z* = 0.03, *p* = 1). In the treatment lineage, no two successive generations were found to be significantly different (GLMs, all *p* > 0.05). The two lineages were not significantly different in any of the generations (GLMs, all *p* > 0.05) (Figure 6B, Table S9).

### 3.6 Fungal Community

**FIGURE 7:**
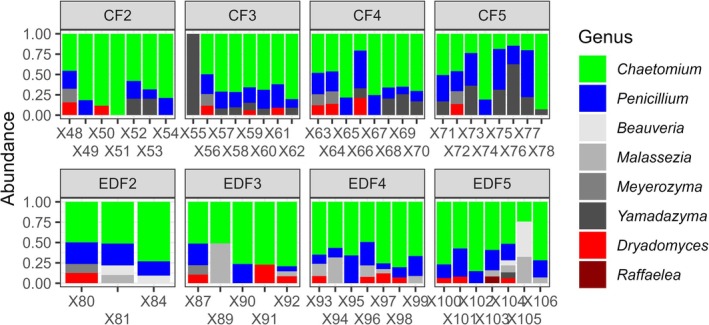
Relative abundances of fungal genera in the two lineages, from F2 to F5. The nutritional symbionts *Dryadomyces* and *Raffaelea* are highlighted in shades of red, while *Chaetomium* and *Penicillium* are highlighted due to their high abundance. Other fungal genera that are less biologically important are represented in shades of gray. The number of nests used for each group is as follows: Control F2 (*N* = 7), F3 (*N* = 8), F4 (*N* = 8), F5 (*N* = 8); Treatment F2 (*N* = 7), F3 (*N* = 6), F4 (*N* = 7), F5 (*N* = 7).

The sequencing run of the 65 samples yielded 1,203,328 raw reads, with a minimum of 145 reads per sample and a maximum of 34,470. The average number of reads per sample was 18,802. After alignment and bioinformatic processing, the resulting dataset contained 59 samples from eight treatment groups, with a total of 1,128,177 raw reads. The number of reads per sample ranged from 9838 to 28,168 (mean = 19,121.64). A total of 70 ASVs were identified at the genus level. The water controls yielded a relatively low number of fungal reads, with a mean of less than 218 reads per sample. In contrast, the artificial medium controls yielded a higher number of reads, reaching levels comparable to those observed in the biological samples. The “decontam” package was used to improve the quality of the dataset and identified a Sordariomycetes and a Lipomyces ASV as contaminants. Accumulation curves of the final dataset, excluding contaminants and control samples, showed that samples approached saturation after approximately 17,000 reads. Visualization of the relative abundance of the genus showed that the groups appeared to be homogeneous (Figure 7).

**FIGURE 8:**
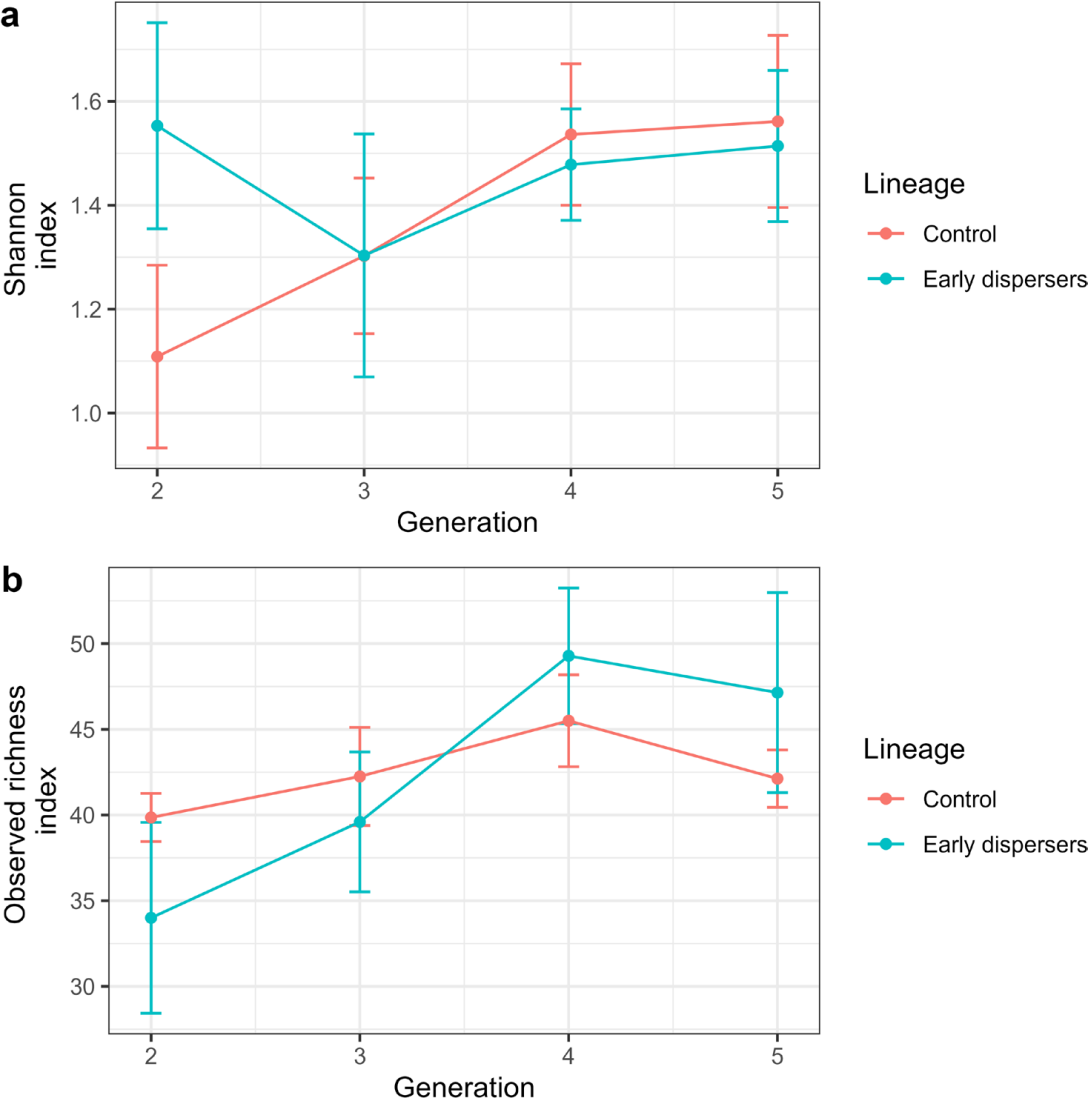
Alpha diversity of the fungal communities of the two lineages, from F2 to F5. (a) Shannon index, (b) Observed richness. The vertical bars show the standard errors of the mean. The number of nests used for each group is as follows: Control: F2 (*N* = 7), F3 (*N* = 8), F4 (*N* = 8), F5 (*N* = 8); Treatment F2 (*N* = 7), F3 (*N* = 6), F4 (*N* = 7), F5 (*N* = 7).

The Shannon diversity index and the observed richness were found to be equal across all generations for each lineage, as well as between lineages for each generation (GLMs, *p* > 0.05). (Figure 8, Tables S10 and S11).

In the control lineage, there was a difference in the beta diversity between F2 and F5 (control F2 vs. F5, PERMANOVA, *F* = 4.04, *p* = 0.035), but no two successive generations were found to be significantly different (PERMANOVA, all *p* > 0.05). In the treatment lineage, there was no difference in beta diversity across generations (PERMANOVA, all *p* > 0.05). A comparison of the two lineages showed that beta diversities differed in F4 and F5 (control F4 vs. treatment F4, PERMANOVA, *F* = 3.11, *p* = 0.003; control F5 vs. treatment F5, PERMANOVA, *F* = 3.94, *p* = 0.02) (Table S12).

## 4 Discussion

The selection experiment was based on pilot results showing variability in the timing of dispersal after five generations. In the pilot experiment, this variability correlated with differences in the success rate, productivity, and social behavior of *X. saxesenii* (Biedermann 2012). It was hypothesized that this variability could result from either inter‐individual plasticity or heritability across generations. However, the study's results do not suggest a heritable element, indicating that the observed variability in the philopatric period is solely explained by inter‐individual plasticity. After five generations of selection, no significant changes in dispersal timing were observed. Therefore, the artificial selection pressure did not produce the expected result. Nevertheless, the data were reported and analyzed. Correlated variables, such as social behavior, productivity, and nest longevity, showed no significant responses between the control and the treatment lineages (Figures 2, 3, 4, 5). The proportion of social behavior exhibited by larvae and adults was found to be similar across all groups (Figure 5), indicating that this aspect of *X. saxesenii* behavior is highly conserved. The inbreeding habits of *X. saxesenii* may result in a lack of variability, preventing an evolutionary response, especially if the selection process starts with a few nests. Previous research has demonstrated that inbreeding and genetic bottlenecks lead to a reduction in adaptive potential (Auld and Relyea 2010; Dierks et al. 2012; Swindell and Bouzat 2005) as well as a general decrease in phenotypic variance (Fowler and Whitlock 1999; Reed et al. 2003).

Interestingly, the majority of the observed changes in the control lineage occurred between the first and second generations. These findings are consistent with other studies that have demonstrated the significant impact of strong selective pressure on the first generations (Irwin and Carter 2014; Tejeda et al. 2016). This suggests that any genetic variability present at the beginning of the selection experiment, if present, was depleted after the first generation.

At the end of the experiment, the beetles in the control lineage dispersed earlier than in the first generation (Figures 2 and 3), which demonstrates variability in this trait. However, this variability may have been selected against by our laboratory conditions. These conditions are favorable for *X. saxesenii* because the breeding system is maintained at a constant temperature, and the artificial medium is nutrient‐rich and free of competing microbes. The benign conditions of the laboratory breeding may have reduced the pressure for cooperative management of beneficial microbes and brood care, resulting in earlier dispersal. Additionally, nest excavation and expansion are much easier in the artificial sawdust medium (held together by agar) than in solid wood, which may have also selected against cooperation. These assumptions are consistent with theoretical models that predict a correlation between harsh environments and increased levels of cooperation (Emlen 1982).

Notably, changes in the fungal community were observed between beetle lineages over time, with significant differences in beta diversity appearing from the fourth generation onwards (Figure 7). The fungal community associated with *X. saxesenii* is complex, and microbial management is critical to the evolution of cooperative behavior (Biedermann and Rohlfs 2017; Nuotclà et al. 2019). Mutualistic fungi, *D. sulphureus* and *R. canadensis*, are conserved and transmitted across generations in the mycetangia (Biedermann et al. 2013; Diehl et al. 2022; Francke‐Grosmann 1975), and are significantly associated with *X. saxesenii* productivity (Biedermann et al. 2013; Nuotclà et al. 2021). The results show that the fungal communities can diverge without significantly affecting *X. saxesenii* . While our primers are effective at detecting these mutualistic fungi, they do not amplify consistently all fungal species, which prevents analysis at the species level. However, superficial data exploration suggested that the abundance of the mutualistic fungi was not strongly affected by our treatment. This suggests that the observed difference in beta diversity is not due to nutritional fungi or important antagonists, but rather to functionally unimportant fungi that do not affect the fitness of the beetles. The divergence of fungal communities may be due to either selection or random drift. Selection would occur when females disperse at different times and transmit different fungal communities. Random drift would result from the repeated transmission bottlenecks to which fungal communities are exposed, as fungal spores are taken up and selectively transmitted in female mycetangia (Mayers et al. 2022).

In several ambrosia beetle species, adult females delay dispersal for variable periods of time and not all reproductive adults leave their natal nest simultaneously (Biedermann, Klepzig, and Taborsky 2011; Nuotclà et al. 2021; Peer and Taborsky 2007). Factors influencing the timing of dispersal are the presence of dependent brood in the nest, the presence of a blocking female in the entrance and changes in air pressure (Biedermann and Taborsky 2011). Also, higher loads of pathogens have been experimentally shown to induce grooming and delay dispersal (Nuotclà et al. 2019). Our results are in line with previous studies on the importance of dependent brood as adult females *X. saxesenii* dispersed earlier in short‐lived and lower productive nests (Figures 2 and 4).

Furthermore, the results indicate that the initial generations are the most responsive to the selection protocol. Early‐dispersing females reproduce more and have a higher direct fitness, while late‐dispersing females cooperate and have a higher indirect fitness (Biedermann, Klepzig, and Taborsky 2011). This suggests that delayed dispersal is an important mechanism in the evolution of the social system of *X. saxesenii* (Peer and Taborsky 2007). However, our results may also be explained by other factors. Laboratory rearing comes with reduced environmental variability and may explain the convergence of the lineages after the initial differences. The reason is that the dispersal of the beetles is affected by environmental conditions, which are homogeneous in our setting. In nature, *X. saxesenii* colonizes wood of different sizes and species, which are exposed to various environmental conditions (sun exposure, humidity, temperature, etc.) (Peer and Taborsky 2007). This leads to highly variable conditions for the growth of its fungi and the development of the brood. Most importantly, some of these habitats may remain stable, providing food for several generations, while others may become unfavorable within the first generation. This is why we assume that the social behavior of this ambrosia beetle species is highly flexible (Kirkendal et al. 2015). Following this argument, the artificial rearing used in this study may favor early dispersal and thus indirectly favor low productivity. Finally, the small number of nests initiated by wild‐trapped females (W5 and W13) and the inbreeding habits of *X. saxesenii* result in a small effective population size. Therefore, generalization must be considered carefully. Currently, it is unclear whether our findings are based on canalized plasticity due to homogeneous laboratory conditions or on missing genetic differences. These factors will be important for future investigations of this species.

*X. saxesenii* is not a conventional model organism and is interesting because of its potential for laboratory rearing, manipulation, and selection. Much remains to be learned about this species, which offers a unique perspective on the evolution of sociality. To understand the evolutionary potential of this species, future research should focus on investigating the genetic variability that exists within and between *X. saxesenii* populations. Of course, we cannot rule out the possibility that selection on the philopatric period may eventually lead to lineages that differ in their social behavior. However, the results so far do not suggest that we would observe more changes if the experiment were longer. Our selection was based on only two nests of wild‐caught individuals whose ancestors showed no signs of a different response. Therefore, it is likely that there was insufficient genetic variability from the outset (our original plan was to start with a much larger population). This remains an important limitation of our study that reduces our ability to draw general conclusions. Replicating the study with a larger initial population would address this issue. Nevertheless, we observed changes between F1 and F2, suggesting that the population initially had some genetic variability. This variability was subsequently lost. Since this may have resulted from laboratory rearing, adjusting the rearing system to avoid selecting for shorter philopatric periods may be worthwhile (e.g., by adding fewer nutrients). Future studies should further investigate the relationship between productivity and philopatric period of *X. saxesenii* . We suggest manipulating the number of adult beetles in the nests to simulate increased dispersal and correlating this manipulation with nest productivity.

## Author Contributions

**Antoine Melet:** conceptualization (equal), data curation (lead), formal analysis (lead), investigation (lead), writing – original draft (lead). **Peter H. W. Biedermann:** conceptualization (equal), funding acquisition (lead), project administration (lead), writing – review and editing (lead).

## Ethics Statement

The authors have nothing to report.

## Conflicts of Interest

The authors declare no conflicts of interest.

## Supporting information


**Appendix S1:** Supporting Information.

## Data Availability

The data and scripts that support the findings of this study are openly available in our GitHub Repository at https://github.com/AntMelet/Artificial‐selection‐on‐X.‐saxesenii.
