# Supplementary material for: Artificial Selection of Early Emerging Helpers in the Cooperatively Breeding Ambrosia Beetle Xyleborinus saxesenii and Its Effects on Various Life History Traits and Their Fungal Symbionts
Source: Ecol Evol. 2025 Oct 20;15(10):e72356. doi: 10.1002/ece3.72356 (PMC12536265; doi:10.1002/ece3.72356)
Supplement: Supplementary file 1 — Appendix S1: Supporting Information. [file ECE3-15-e72356-s001.docx]

| Groups | **wild-caught** | **F0** | **CF1** | **CF2** | **CF3** | **CF4** | **CF5** |
| --- | --- | --- | --- | --- | --- | --- | --- |
| Number of nests | 2 | 5 | 7 | 8 | 8 | 15 | 76 |
|  | W13 | 99 | CF1-350 | CF2-449 | CF3-87 | CF4-128 | CF5-151 |
|  | W13 | 99 | CF1-350 | CF2-449 | CF3-87 | CF4-128 | CF5-184 |
|  | W13 | 99 | CF1-350 | CF2-449 | CF3-87 | CF4-128 | CF5-186 |
|  | W5 | 115 | CF1-261 | CF2-513 | CF3-158 | CF4-271 | CF5-359 |
|  | W5 | 115 | CF1-300 | CF2-358 | CF3-40 | CF4-60 | CF5-112 |
|  | W5 | 115 | CF1-300 | CF2-358 | CF3-40 | CF4-60 | CF5-132 |
|  | W5 | 115 | CF1-300 | CF2-358 | CF3-40 | CF4-60 | CF5-154 |
|  | W5 | 115 | CF1-300 | CF2-358 | CF3-40 | CF4-61 | CF5-110 |
|  | W5 | 115 | CF1-300 | CF2-358 | CF3-40 | CF4-62 | CF5-191 |
|  | W5 | 115 | CF1-300 | CF2-358 | CF3-40 | CF4-62 | CF5-192 |
|  | W5 | 115 | CF1-300 | CF2-358 | CF3-40 | CF4-62 | CF5-194 |
|  | W5 | 115 | CF1-300 | CF2-358 | CF3-40 | CF4-62 | CF5-195 |
|  | W5 | 115 | CF1-300 | CF2-358 | CF3-40 | CF4-62 | CF5-196 |
|  | W5 | 115 | CF1-300 | CF2-358 | CF3-40 | CF4-62 | CF5-197 |
|  | W5 | 115 | CF1-300 | CF2-358 | CF3-40 | CF4-62 | CF5-199 |
|  | W5 | 115 | CF1-300 | CF2-358 | CF3-40 | CF4-62 | CF5-200 |
|  | W5 | 115 | CF1-300 | CF2-358 | CF3-40 | CF4-62 | CF5-201 |
|  | W5 | 115 | CF1-300 | CF2-358 | CF3-40 | CF4-62 | CF5-202 |
|  | W5 | 115 | CF1-300 | CF2-358 | CF3-40 | CF4-62 | CF5-203 |
|  | W5 | 115 | CF1-300 | CF2-358 | CF3-40 | CF4-62 | CF5-204 |
|  | W5 | 115 | CF1-300 | CF2-358 | CF3-40 | CF4-62 | CF5-206 |
|  | W5 | 115 | CF1-300 | CF2-358 | CF3-40 | CF4-64 | CF5-119 |
|  | W5 | 148 | CF1-166 | CF2-265 | CF3-17 | CF4-12 | CF5-14 |
|  | W5 | 148 | CF1-166 | CF2-265 | CF3-17 | CF4-12 | CF5-16 |
|  | W5 | 148 | CF1-166 | CF2-265 | CF3-17 | CF4-12 | CF5-45 |
|  | W5 | 148 | CF1-166 | CF2-265 | CF3-17 | CF4-12 | CF5-50 |
|  | W5 | 148 | CF1-166 | CF2-265 | CF3-17 | CF4-12 | CF5-51 |
|  | W5 | 148 | CF1-166 | CF2-265 | CF3-17 | CF4-12 | CF5-52 |
|  | W13 | 166 | CF1-270 | CF2-298 | CF3-45 | CF4-78 | CF5-136 |
|  | W13 | 166 | CF1-270 | CF2-298 | CF3-45 | CF4-78 | CF5-137 |
|  | W13 | 166 | CF1-270 | CF2-298 | CF3-45 | CF4-78 | CF5-138 |
|  | W13 | 166 | CF1-270 | CF2-298 | CF3-45 | CF4-78 | CF5-139 |
|  | W13 | 166 | CF1-270 | CF2-298 | CF3-45 | CF4-78 | CF5-140 |
|  | W13 | 166 | CF1-274 | CF2-306 | CF3-49 | CF4-142 | CF5-187 |
|  | W13 | 166 | CF1-274 | CF2-306 | CF3-49 | CF4-142 | CF5-232 |
|  | W13 | 166 | CF1-274 | CF2-306 | CF3-49 | CF4-142 | CF5-234 |
|  | W13 | 166 | CF1-274 | CF2-306 | CF3-49 | CF4-142 | CF5-235 |
|  | W13 | 166 | CF1-274 | CF2-306 | CF3-49 | CF4-142 | CF5-240 |
|  | W13 | 166 | CF1-274 | CF2-306 | CF3-49 | CF4-142 | CF5-242 |
|  | W13 | 166 | CF1-274 | CF2-306 | CF3-49 | CF4-142 | CF5-261 |
|  | W13 | 166 | CF1-274 | CF2-306 | CF3-49 | CF4-142 | CF5-262 |
|  | W13 | 166 | CF1-274 | CF2-306 | CF3-49 | CF4-142 | CF5-263 |
|  | W13 | 166 | CF1-274 | CF2-306 | CF3-49 | CF4-142 | CF5-264 |
|  | W13 | 166 | CF1-274 | CF2-306 | CF3-49 | CF4-142 | CF5-265 |
|  | W13 | 166 | CF1-274 | CF2-306 | CF3-49 | CF4-142 | CF5-269 |
|  | W13 | 166 | CF1-274 | CF2-306 | CF3-49 | CF4-142 | CF5-270 |
|  | W13 | 166 | CF1-274 | CF2-306 | CF3-49 | CF4-142 | CF5-271 |
|  | W13 | 166 | CF1-274 | CF2-306 | CF3-49 | CF4-142 | CF5-272 |
|  | W13 | 166 | CF1-274 | CF2-306 | CF3-49 | CF4-155 | CF5-243 |
|  | W13 | 166 | CF1-274 | CF2-306 | CF3-49 | CF4-155 | CF5-245 |
|  | W13 | 166 | CF1-274 | CF2-306 | CF3-49 | CF4-155 | CF5-247 |
|  | W13 | 166 | CF1-274 | CF2-306 | CF3-49 | CF4-155 | CF5-274 |
|  | W13 | 166 | CF1-274 | CF2-306 | CF3-49 | CF4-156 | CF5-224 |
|  | W13 | 166 | CF1-274 | CF2-306 | CF3-49 | CF4-156 | CF5-249 |
|  | W13 | 166 | CF1-274 | CF2-306 | CF3-49 | CF4-156 | CF5-253 |
|  | W13 | 166 | CF1-274 | CF2-306 | CF3-49 | CF4-156 | CF5-254 |
|  | W13 | 166 | CF1-274 | CF2-308 | CF3-54 | CF4-124 | CF5-335 |
|  | W13 | 166 | CF1-274 | CF2-308 | CF3-54 | CF4-124 | CF5-336 |
|  | W13 | 166 | CF1-274 | CF2-308 | CF3-54 | CF4-124 | CF5-337 |
|  | W13 | 166 | CF1-274 | CF2-308 | CF3-54 | CF4-124 | CF5-339 |
|  | W13 | 166 | CF1-274 | CF2-308 | CF3-54 | CF4-124 | CF5-340 |
|  | W13 | 166 | CF1-274 | CF2-308 | CF3-54 | CF4-124 | CF5-343 |
|  | W13 | 166 | CF1-274 | CF2-308 | CF3-54 | CF4-124 | CF5-347 |
|  | W5 | 237 | CF1-316 | CF2-383 | CF3-77 | CF4-166 | CF5-190 |
|  | W5 | 237 | CF1-316 | CF2-383 | CF3-77 | CF4-166 | CF5-282 |
|  | W5 | 237 | CF1-316 | CF2-383 | CF3-77 | CF4-166 | CF5-283 |
|  | W5 | 237 | CF1-316 | CF2-383 | CF3-77 | CF4-166 | CF5-305 |
|  | W5 | 237 | CF1-316 | CF2-383 | CF3-77 | CF4-168 | CF5-286 |
|  | W5 | 237 | CF1-316 | CF2-383 | CF3-77 | CF4-168 | CF5-289 |
|  | W5 | 237 | CF1-316 | CF2-383 | CF3-77 | CF4-168 | CF5-290 |
|  | W5 | 237 | CF1-316 | CF2-383 | CF3-77 | CF4-168 | CF5-291 |
|  | W5 | 237 | CF1-316 | CF2-383 | CF3-77 | CF4-168 | CF5-307 |
|  | W5 | 237 | CF1-316 | CF2-383 | CF3-77 | CF4-190 | CF5-309 |
|  | W5 | 237 | CF1-316 | CF2-383 | CF3-77 | CF4-190 | CF5-310 |
|  | W5 | 237 | CF1-316 | CF2-383 | CF3-77 | CF4-190 | CF5-311 |
|  | W5 | 237 | CF1-316 | CF2-383 | CF3-77 | CF4-190 | CF5-327 |
|  |  |  |  |  |  |  |  |
|  |  |  |  |  |  |  |  |
|  |  |  |  |  |  |  |  |
|  |  |  |  |  |  |  |  |
|  |  |  |  |  |  |  |  |
|  |  |  |  |  |  |  |  |
|  |  |  |  |  |  |  |  |
|  |  |  |  |  |  |  |  |
|  |  |  |  |  |  |  |  |
|  |  |  |  |  |  |  |  |
|  |  |  |  |  |  |  |  |
| Groups | **wild-caught** | **F0** | **EDF1** | **EDF2** | **EDF3** | **EDF4** | **EDF5** |
| Number of nests | 2 | 5 | 7 | 9 | 14 | 23 | 86 |
|  | W5 | 41 | EDF1-5 | EDF2-4 | EDF3-7 | EDF4-21 | EDF5-20 |
|  | W5 | 41 | EDF1-5 | EDF2-4 | EDF3-9 | EDF4-11 | EDF5-6 |
|  | W5 | 41 | EDF1-5 | EDF2-4 | EDF3-9 | EDF4-11 | EDF5-7 |
|  | W5 | 41 | EDF1-5 | EDF2-4 | EDF3-9 | EDF4-12 | EDF5-17 |
|  | W5 | 41 | EDF1-5 | EDF2-4 | EDF3-9 | EDF4-22 | EDF5-13 |
|  | W5 | 41 | EDF1-5 | EDF2-4 | EDF3-9 | EDF4-22 | EDF5-14 |
|  | W5 | 41 | EDF1-5 | EDF2-4 | EDF3-9 | EDF4-22 | EDF5-15 |
|  | W5 | 41 | EDF1-5 | EDF2-4 | EDF3-9 | EDF4-22 | EDF5-16 |
|  | W5 | 41 | EDF1-5 | EDF2-4 | EDF3-9 | EDF4-22 | EDF5-21 |
|  | W5 | 106 | EDF1-50 | EDF2-65 | EDF3-48 | EDF4-37 | EDF5-22 |
|  | W5 | 106 | EDF1-50 | EDF2-65 | EDF3-48 | EDF4-37 | EDF5-25 |
|  | W5 | 106 | EDF1-50 | EDF2-65 | EDF3-48 | EDF4-37 | EDF5-35 |
|  | W5 | 106 | EDF1-50 | EDF2-65 | EDF3-48 | EDF4-37 | EDF5-36 |
|  | W5 | 106 | EDF1-50 | EDF2-65 | EDF3-48 | EDF4-37 | EDF5-37 |
|  | W5 | 106 | EDF1-50 | EDF2-65 | EDF3-48 | EDF4-37 | EDF5-38 |
|  | W5 | 106 | EDF1-50 | EDF2-65 | EDF3-48 | EDF4-38 | EDF5-27 |
|  | W5 | 106 | EDF1-50 | EDF2-65 | EDF3-48 | EDF4-38 | EDF5-40 |
|  | W5 | 106 | EDF1-50 | EDF2-65 | EDF3-48 | EDF4-38 | EDF5-41 |
|  | W5 | 106 | EDF1-50 | EDF2-65 | EDF3-48 | EDF4-38 | EDF5-44 |
|  | W5 | 106 | EDF1-50 | EDF2-65 | EDF3-48 | EDF4-38 | EDF5-45 |
|  | W5 | 106 | EDF1-50 | EDF2-65 | EDF3-48 | EDF4-38 | EDF5-47 |
|  | W5 | 106 | EDF1-50 | EDF2-65 | EDF3-52 | EDF4-41 | EDF5-33 |
|  | W5 | 106 | EDF1-50 | EDF2-65 | EDF3-52 | EDF4-41 | EDF5-42 |
|  | W5 | 106 | EDF1-50 | EDF2-65 | EDF3-52 | EDF4-41 | EDF5-43 |
|  | W5 | 129 | EDF1-73 | EDF2-95 | EDF3-94 | EDF4-121 | EDF5-102 |
|  | W5 | 129 | EDF1-73 | EDF2-95 | EDF3-94 | EDF4-121 | EDF5-103 |
|  | W5 | 129 | EDF1-73 | EDF2-95 | EDF3-94 | EDF4-121 | EDF5-104 |
|  | W5 | 129 | EDF1-73 | EDF2-95 | EDF3-94 | EDF4-121 | EDF5-105 |
|  | W5 | 129 | EDF1-73 | EDF2-95 | EDF3-94 | EDF4-121 | EDF5-106 |
|  | W5 | 129 | EDF1-73 | EDF2-95 | EDF3-94 | EDF4-122 | EDF5-107 |
|  | W5 | 129 | EDF1-73 | EDF2-95 | EDF3-94 | EDF4-122 | EDF5-154 |
|  | W5 | 129 | EDF1-73 | EDF2-95 | EDF3-94 | EDF4-122 | EDF5-155 |
|  | W5 | 129 | EDF1-73 | EDF2-95 | EDF3-94 | EDF4-143 | EDF5-132 |
|  | W5 | 129 | EDF1-73 | EDF2-95 | EDF3-94 | EDF4-143 | EDF5-133 |
|  | W5 | 129 | EDF1-73 | EDF2-95 | EDF3-94 | EDF4-143 | EDF5-134 |
|  | W5 | 129 | EDF1-73 | EDF2-95 | EDF3-94 | EDF4-143 | EDF5-135 |
|  | W5 | 129 | EDF1-73 | EDF2-95 | EDF3-95 | EDF4-123 | EDF5-110 |
|  | W5 | 129 | EDF1-73 | EDF2-95 | EDF3-95 | EDF4-123 | EDF5-111 |
|  | W5 | 129 | EDF1-73 | EDF2-95 | EDF3-95 | EDF4-123 | EDF5-158 |
|  | W5 | 129 | EDF1-73 | EDF2-95 | EDF3-95 | EDF4-123 | EDF5-159 |
|  | W5 | 129 | EDF1-73 | EDF2-95 | EDF3-95 | EDF4-123 | EDF5-161 |
|  | W5 | 129 | EDF1-75 | EDF2-99 | EDF3-96 | EDF4-150 | EDF5-136 |
|  | W5 | 129 | EDF1-75 | EDF2-99 | EDF3-96 | EDF4-150 | EDF5-137 |
|  | W5 | 129 | EDF1-75 | EDF2-102 | EDF3-73 | EDF4-103 | EDF5-97 |
|  | W5 | 129 | EDF1-75 | EDF2-102 | EDF3-73 | EDF4-137 | EDF5-126 |
|  | W5 | 129 | EDF1-75 | EDF2-102 | EDF3-73 | EDF4-137 | EDF5-162 |
|  | W5 | 129 | EDF1-75 | EDF2-102 | EDF3-102 | EDF4-129 | EDF5-112 |
|  | W5 | 129 | EDF1-75 | EDF2-102 | EDF3-102 | EDF4-129 | EDF5-113 |
|  | W5 | 129 | EDF1-75 | EDF2-102 | EDF3-102 | EDF4-129 | EDF5-114 |
|  | W5 | 129 | EDF1-75 | EDF2-102 | EDF3-102 | EDF4-129 | EDF5-115 |
|  | W5 | 129 | EDF1-75 | EDF2-102 | EDF3-102 | EDF4-129 | EDF5-116 |
|  | W5 | 129 | EDF1-75 | EDF2-102 | EDF3-102 | EDF4-129 | EDF5-118 |
|  | W5 | 129 | EDF1-75 | EDF2-102 | EDF3-102 | EDF4-131 | EDF5-119 |
|  | W5 | 129 | EDF1-75 | EDF2-102 | EDF3-102 | EDF4-131 | EDF5-121 |
|  | W5 | 129 | EDF1-75 | EDF2-102 | EDF3-102 | EDF4-131 | EDF5-123 |
|  | W5 | 129 | EDF1-75 | EDF2-102 | EDF3-102 | EDF4-131 | EDF5-124 |
|  | W5 | 129 | EDF1-75 | EDF2-102 | EDF3-102 | EDF4-131 | EDF5-125 |
|  | W5 | 149 | EDF1-78 | EDF2-109 | EDF3-67 | EDF4-90 | EDF5-87 |
|  | W5 | 149 | EDF1-78 | EDF2-109 | EDF3-67 | EDF4-90 | EDF5-90 |
|  | W5 | 149 | EDF1-78 | EDF2-109 | EDF3-67 | EDF4-90 | EDF5-91 |
|  | W5 | 149 | EDF1-78 | EDF2-109 | EDF3-67 | EDF4-90 | EDF5-92 |
|  | W5 | 149 | EDF1-79 | EDF2-88 | EDF3-86 | EDF4-142 | EDF5-128 |
|  | W5 | 149 | EDF1-79 | EDF2-89 | EDF3-88 | EDF4-110 | EDF5-98 |
|  | W5 | 149 | EDF1-79 | EDF2-89 | EDF3-88 | EDF4-110 | EDF5-99 |
|  | W5 | 149 | EDF1-79 | EDF2-89 | EDF3-88 | EDF4-110 | EDF5-100 |
|  | W5 | 149 | EDF1-79 | EDF2-89 | EDF3-88 | EDF4-110 | EDF5-101 |
|  | W13 | 154 | EDF1-64 | EDF2-63 | EDF3-60 | EDF4-87 | EDF5-75 |
|  | W13 | 154 | EDF1-64 | EDF2-63 | EDF3-60 | EDF4-87 | EDF5-76 |
|  | W13 | 154 | EDF1-64 | EDF2-63 | EDF3-60 | EDF4-100 | EDF5-95 |
|  | W13 | 154 | EDF1-64 | EDF2-63 | EDF3-60 | EDF4-100 | EDF5-96 |
|  | W13 | 154 | EDF1-64 | EDF2-63 | EDF3-60 | EDF4-100 | EDF5-140 |
|  | W13 | 154 | EDF1-64 | EDF2-63 | EDF3-60 | EDF4-100 | EDF5-141 |
|  | W13 | 154 | EDF1-64 | EDF2-63 | EDF3-62 | EDF4-48 | EDF5-65 |
|  | W13 | 154 | EDF1-64 | EDF2-63 | EDF3-62 | EDF4-48 | EDF5-66 |
|  | W13 | 154 | EDF1-64 | EDF2-63 | EDF3-62 | EDF4-48 | EDF5-67 |
|  | W13 | 154 | EDF1-64 | EDF2-63 | EDF3-62 | EDF4-48 | EDF5-70 |
|  | W13 | 154 | EDF1-64 | EDF2-63 | EDF3-62 | EDF4-48 | EDF5-71 |
|  | W13 | 154 | EDF1-64 | EDF2-63 | EDF3-62 | EDF4-64 | EDF5-50 |
|  | W13 | 154 | EDF1-64 | EDF2-63 | EDF3-62 | EDF4-64 | EDF5-51 |
|  | W13 | 154 | EDF1-64 | EDF2-63 | EDF3-62 | EDF4-64 | EDF5-52 |
|  | W13 | 154 | EDF1-64 | EDF2-63 | EDF3-62 | EDF4-64 | EDF5-53 |
|  | W13 | 154 | EDF1-64 | EDF2-63 | EDF3-62 | EDF4-64 | EDF5-54 |
|  | W13 | 154 | EDF1-64 | EDF2-63 | EDF3-62 | EDF4-64 | EDF5-55 |
|  | W13 | 154 | EDF1-64 | EDF2-63 | EDF3-62 | EDF4-64 | EDF5-56 |
|  | W13 | 154 | EDF1-64 | EDF2-63 | EDF3-62 | EDF4-64 | EDF5-72 |
|  | W13 | 154 | EDF1-64 | EDF2-63 | EDF3-62 | EDF4-64 | EDF5-73 |

*Supplementary table 1*: Genealogy of the nests used for life history and behavioural analysis. The first part of the table contains information about the nests used in the control groups. The second part of the table contains information about the nests used in the treatment groups. For each part, the first line indicates the names of the groups considered. The second line indicates the number of different nests the group contains. The following lines each indicate the genealogy of one nest of generation F5. The genealogy reads from left to right, each change of cell indicates the parent and offspring colonies. It continues to the final generation of the experiment.

| Groups | **wild-caught** | **F0** | **CF1** | **CF2** | **CF3** | **CF4** | **CF5** |
| --- | --- | --- | --- | --- | --- | --- | --- |
|  | W5 | 148 | CF1-166 | CF2-261 |  |  |  |
|  | W13 | 166 | CF1-270 | CF2-299 |  |  |  |
|  | W13 | 166 | CF1-274 | CF2-312 |  |  |  |
|  | W13 | 166 | CF1-274 | CF2-314 |  |  |  |
|  | W5 | 115 | CF1-305 | CF2-423 |  |  |  |
|  | W5 | 115 | CF1-305 | CF2-425 |  |  |  |
|  | W5 | 115 | CF1-333 | CF2-587 |  |  |  |
|  | W5 | 148 | CF1-166 | CF2-266 | CF3-19 |  |  |
|  | W13 | 166 | CF1-274 | CF2-325 | CF3-56 |  |  |
|  | W13 | 166 | CF1-274 | CF2-325 | CF3-66 |  |  |
|  | W5 | 115 | CF1-306 | CF2-493 | CF3-122 |  |  |
|  | W5 | 115 | CF1-306 | CF2-525 | CF3-180 |  |  |
|  | W5 | 115 | CF1-333 | CF2-608 | CF3-212 |  |  |
|  | W5 | 115 | CF1-333 | CF2-607 | CF3-214 |  |  |
|  | W5 | 115 | CF1-333 | CF2-607 | CF3-228 |  |  |
|  | W13 | 166 | CF1-270 | CF2-298 | CF3-45 | CF4-80 |  |
|  | W13 | 99 | CF1-350 | CF2-449 | CF3-87 | CF4-100 |  |
|  | W13 | 166 | CF1-274 | CF2-306 | CF3-49 | CF4-119 |  |
|  | W13 | 166 | CF1-274 | CF2-308 | CF3-54 | CF4-123 |  |
|  | W13 | 99 | CF1-350 | CF2-449 | CF3-87 | CF4-130 |  |
|  | W5 | 237 | CF1-316 | CF2-383 | CF3-77 | CF4-170 |  |
|  | W5 | 237 | CF1-316 | CF2-383 | CF3-77 | CF4-176 |  |
|  | W5 | 115 | CF1-306 | CF2-497 | CF3-116 | CF4-218 |  |
|  | W5 | 115 | CF1-300 | CF2-358 | CF3-40 | CF4-64 | CF5-119 |
|  | W13 | 166 | CF1-274 | CF2-306 | CF3-51 | CF4-97 | CF5-171 |
|  | W13 | 166 | CF1-274 | CF2-306 | CF3-49 | CF4-142 | CF5-235 |
|  | W13 | 166 | CF1-274 | CF2-306 | CF3-49 | CF4-156 | CF5-254 |
|  | W13 | 166 | CF1-274 | CF2-306 | CF3-49 | CF4-142 | CF5-263 |
|  | W13 | 166 | CF1-274 | CF2-306 | CF3-49 | CF4-142 | CF5-270 |
|  | W13 | 166 | CF1-274 | CF2-306 | CF3-49 | CF4-142 | CF5-271 |
|  | W5 | 237 | CF1-316 | CF2-383 | CF3-77 | CF4-168 | CF5-286 |
|  |  |  |  |  |  |  |  |
|  |  |  |  |  |  |  |  |
|  |  |  |  |  |  |  |  |
|  |  |  |  |  |  |  |  |
|  |  |  |  |  |  |  |  |
|  |  |  |  |  |  |  |  |
|  |  |  |  |  |  |  |  |
|  |  |  |  |  |  |  |  |
|  |  |  |  |  |  |  |  |
|  |  |  |  |  |  |  |  |
|  |  |  |  |  |  |  |  |
|  |  |  |  |  |  |  |  |
|  |  |  |  |  |  |  |  |
|  |  |  |  |  |  |  |  |
|  |  |  |  |  |  |  |  |
|  |  |  |  |  |  |  |  |
|  |  |  |  |  |  |  |  |
|  |  |  |  |  |  |  |  |
|  |  |  |  |  |  |  |  |
|  |  |  |  |  |  |  |  |
|  |  |  |  |  |  |  |  |
|  |  |  |  |  |  |  |  |
|  |  |  |  |  |  |  |  |
|  |  |  |  |  |  |  |  |
|  |  |  |  |  |  |  |  |
| Groups | **wild-caught** | **F0** | **EDF1** | **EDF2** | **EDF3** | **EDF4** | **EDF5** |
|  | W5 | 41 | EDF1-16 | EDF2-19 |  |  |  |
|  | W5 | 41 | EDF1-16 | EDF2-20 |  |  |  |
|  | W13 | 154 | EDF1-64 | EDF2-69 |  |  |  |
|  | W5 | 41 | EDF1-5 | EDF2-4 | EDF3-1 |  |  |
|  | W5 | 106 | EDF1-50 | EDF2-65 | EDF3-53 |  |  |
|  | W5 | 41 | EDF1-16 | EDF2-18 | EDF3-55 |  |  |
|  | W5 | 129 | EDF1-75 | EDF2-99 | EDF3-82 |  |  |
|  | W5 | 149 | EDF1-79 | EDF2-89 | EDF3-89 |  |  |
|  | W5 | 41 | EDF1-5 | EDF2-4 | EDF3-7 | EDF4-20 |  |
|  | W5 | 41 | EDF1-5 | EDF2-4 | EDF3-7 | EDF4-21 |  |
|  | W5 | 106 | EDF1-50 | EDF2-65 | EDF3-48 | EDF4-35 |  |
|  | W5 | 149 | EDF1-79 | EDF2-85 | EDF3-65 | EDF4-53 |  |
|  | W5 | 149 | EDF1-78 | EDF2-109 | EDF3-67 | EDF4-70 |  |
|  | W5 | 129 | EDF1-75 | EDF2-102 | EDF3-73 | EDF4-136 |  |
|  | W5 | 129 | EDF1-73 | EDF2-95 | EDF3-94 | EDF4-144 |  |
|  | W5 | 106 | EDF1-50 | EDF2-65 | EDF3-48 | EDF4-37 | EDF5-22 |
|  | W5 | 106 | EDF1-50 | EDF2-65 | EDF3-48 | EDF4-37 | EDF5-38 |
|  | W5 | 106 | EDF1-50 | EDF2-65 | EDF3-48 | EDF4-38 | EDF5-41 |
|  | W5 | 41 | EDF1-16 | EDF2-18 | EDF3-74 | EDF4-72 | EDF5-60 |
|  | W5 | 149 | EDF1-78 | EDF2-109 | EDF3-102 | EDF4-129 | EDF5-118 |
|  | W5 | 149 | EDF1-78 | EDF2-109 | EDF3-102 | EDF4-131 | EDF5-124 |
|  | W5 | 129 | EDF1-75 | EDF2-99 | EDF3-96 | EDF4-150 | EDF5-137 |

*Supplementary table 2*: Genealogy of the nests used for life history and behavioural analysis. The first part of the table contains information about the nests used in the control groups. The second part of the table contains information about the nests used in the treatment groups. For each part, the first line indicates the names of the groups considered. The following lines each indicate the genealogy of one nest. The genealogy reads from left to right, each change of cell indicates the parent and offspring colonies. It continues to the colonies that were used for the data collection.

| Comparison between groups | z value | p-value |
| --- | --- | --- |
| CF1 – CF5 | -4.21 | < 0.01 |
| CF1 – CF2 | -3.47 | 0.02 |
| CF2 – CF3 | 2.53 | 0.23 |
| CF3 – CF4 | -1.38 | 0.92 |
| CF4 – CF5 | -1.65 | 0.8 |
| EDF1 – EDF5 | 0.07 | 1 |
| EDF1 – EDF2 | -0.06 | 1 |
| EDF2 – EDF3 | 0.63 | 1 |
| EDF3 – EDF4 | 0.48 | 1 |
| EDF4 – EDF5 | -1.6 | 0.83 |
| CF1 – EDF1 | -2.7 | 0.16 |
| CF2 – EDF2 | 0.66 | 1 |
| CF3 – EDF3 | -1.54 | 0.86 |
| CF4 – EDF4 | 0.25 | 1 |
| CF5 – EDF5 | 1.09 | 0.98 |

Supplementary table 3: Summary of the multi-level generalized model used to compare the lifespan of the different groups.

| Comparison between groups | z value | p-value |
| --- | --- | --- |
| CF1 – CF5 | -1.68 | 0.78 |
| CF1 – CF2 | -1.27 | 0.95 |
| CF2 – CF3 | 0.24 | 1 |
| CF3 – CF4 | -0.23 | 1 |
| CF4 – CF5 | -0.06 | 1 |
| EDF1 – EDF5 | -0.24 | 1 |
| EDF1 – EDF2 | -0.89 | 1 |
| EDF2 – EDF3 | 1.2 | 0.97 |
| EDF3 – EDF4 | 0.37 | 1 |
| EDF4 – EDF5 | -1.23 | 0.96 |
| CF1 – EDF1 | -0.8 | 1 |
| CF2 – EDF2 | -0.45 | 1 |
| CF3 – EDF3 | 0.4 | 1 |
| CF4 – EDF4 | 1.21 | 0.97 |
| CF5 – EDF5 | 0.84 | 1 |

Supplementary table 4: Summary of the multi-level generalized model used to compare the time before the start of dispersal of the different groups.

| Comparison between groups | z value | p-value |
| --- | --- | --- |
| CF1 – CF5 | -15.27 | < 0.01 |
| CF1 – CF2 | -9.38 | < 0.01 |
| CF2 – CF3 | 5.04 | < 0.01 |
| CF3 – CF4 | 0.2 | 1 |
| CF4 – CF5 | -9 | < 0.01 |
| EDF1 – EDF5 | 0.29 | 1 |
| EDF1 – EDF2 | -1.36 | 0.93 |
| EDF2 – EDF3 | 4.49 | < 0.01 |
| EDF3 – EDF4 | 1.65 | 0.8 |
| EDF4 – EDF5 | -8.17 | < 0.01 |
| CF1 – EDF1 | -5.97 | < 0.01 |
| CF2 – EDF2 | 1.91 | 0.63 |
| CF3 – EDF3 | 1.33 | 0.94 |
| CF4 – EDF4 | 3.24 | 0.03 |
| CF5 – EDF5 | 9.03 | < 0.01 |

Supplementary table 5: Summary of the multi-level generalized model used to compare the productivity of the different groups.

| Comparison between groups | z value | p-value |
| --- | --- | --- |
| CF1 – CF5 | -3.42 | 0.02 |
| CF1 – CF2 | -0.38 | 1 |
| CF2 – CF3 | 0.22 | 1 |
| CF3 – CF4 | -2.57 | 0.2 |
| CF4 – CF5 | 0.13 | 1 |
| EDF1 – EDF5 | -1.49 | 0.87 |
| EDF1 – EDF2 | 0.69 | 1 |
| EDF2 – EDF3 | -0.76 | 1 |
| EDF3 – EDF4 | -1.61 | 0.81 |
| EDF4 – EDF5 | -1.39 | 0.91 |
| CF1 – EDF1 | -1.05 | 0.99 |
| CF2 – EDF2 | 0.23 | 1 |
| CF3 – EDF3 | -1.09 | 0.98 |
| CF4 – EDF4 | -0.12 | 1 |
| CF5 – EDF5 | -2.15 | 0.44 |

Supplementary table 6: Summary of the multi-level generalized model used to compare the social behaviour of larvae of the different groups.

| Comparison between groups | z value | p-value |
| --- | --- | --- |
| CF1 – CF5 | -2.45 | 0.26 |
| CF1 – CF2 | -2.4 | 0.29 |
| CF2 – CF3 | 2.26 | 0.37 |
| CF3 – CF4 | -2.16 | 0.43 |
| CF4 – CF5 | 0.97 | 0.99 |
| EDF1 – EDF5 | -0.21 | 1 |
| EDF1 – EDF2 | -0.27 | 1 |
| EDF2 – EDF3 | -0.28 | 1 |
| EDF3 – EDF4 | -0.02 | 1 |
| EDF4 – EDF5 | 0.91 | 1 |
| CF1 – EDF1 | -1.04 | 0.99 |
| CF2 – EDF2 | 1.58 | 0.83 |
| CF3 – EDF3 | -1.77 | 0.71 |
| CF4 – EDF4 | 0.07 | 1 |
| CF5 – EDF5 | -0.28 | 1 |

Supplementary table 7: Summary of the multi-level generalized model used to compare the social behaviour of adults of the different groups.

| Comparison between groups | z value | p-value |
| --- | --- | --- |
| CF1 – CF5 | 4.74 | < 0.01 |
| CF1 – CF2 | 4.24 | < 0.01 |
| CF2 – CF3 | -3.08 | 0.05 |
| CF3 – CF4 | 1 | 0.99 |
| CF4 – CF5 | 0.8 | 1 |
| EDF1 – EDF5 | 2.66 | 0.16 |
| EDF1 – EDF2 | 1.77 | 0.71 |
| EDF2 – EDF3 | -0.25 | 1 |
| EDF3 – EDF4 | -3.12 | 0.05 |
| EDF4 – EDF5 | 5.75 | < 0.01 |
| CF1 – EDF1 | 0.34 | 1 |
| CF2 – EDF2 | -2.22 | 0.39 |
| CF3 – EDF3 | 0.92 | 0.99 |
| CF4 – EDF4 | -3.53 | 0.01 |
| CF5 – EDF5 | 0.56 | 1 |

Supplementary table 8: Summary of the multi-level generalized model used to compare the activity of larvae of the different groups.

| Comparison between groups | Test statistic | p-value |
| --- | --- | --- |
| CF1 – CF5 | 3.95 | < 0.01 |
| CF1 – CF2 | 3.8 | < 0.01 |
| CF2 – CF3 | -2.97 | 0.07 |
| CF3 – CF4 | 0.31 | 1 |
| CF4 – CF5 | 2.09 | 0.49 |
| EDF1 – EDF5 | < 0.01 | 1 |
| EDF1 – EDF2 | 0.92 | 0.99 |
| EDF2 – EDF3 | -2.59 | 0.19 |
| EDF3 – EDF4 | 2.13 | 0.46 |
| EDF4 – EDF5 | -0.73 | 1 |
| CF1 – EDF1 | 1.04 | 0.99 |
| CF2 – EDF2 | -1.56 | 0.85 |
| CF3 – EDF3 | -1.09 | 0.98 |
| CF4 – EDF4 | 0.76 | 1 |
| CF5 – EDF5 | -2.84 | 0.11 |

Supplementary table 9: Summary of the multi-level generalized model used to compare the activity of adults of the different groups.

| Comparison between groups | z value | p-value |
| --- | --- | --- |
| CF2 – CF5 | 2.1 | 0.41 |
| CF2 – CF3 | 0.9 | 0.99 |
| CF3 – CF4 | 1.12 | 0.95 |
| CF4 – CF5 | 0.12 | 1 |
| EDF2 – EDF5 | -0.14 | 1 |
| EDF2 – EDF3 | -0.82 | 0.99 |
| EDF3 – EDF4 | 0.72 | 1 |
| EDF4 – EDF5 | 0.16 | 1 |
| CF2 – EDF2 | 1.54 | 0.78 |
| CF3 – EDF3 | < 0.01 | 1 |
| CF4 – EDF4 | -0.27 | 1 |
| CF5 – EDF5 | -0.22 | 1 |

Supplementary table 10: Summary of the multi-level generalized model used to compare the Shannon index of the fugal community of the different groups.

| Comparison between groups | z value | p-value |
| --- | --- | --- |
| CF2 – CF5 | 0.48 | 1 |
| CF2 – CF3 | 0.51 | 1 |
| CF3 – CF4 | 0.72 | 1 |
| CF4 – CF5 | -0.74 | 1 |
| EDF2 – EDF5 | 2.1 | 0.41 |
| EDF2 – EDF3 | 0.85 | 0.99 |
| EDF3 – EDF4 | 1.82 | 0.6 |
| EDF4 – EDF5 | -0.44 | 1 |
| CF2 – EDF2 | -0.94 | 0.98 |
| CF3 – EDF3 | -0.51 | 1 |
| CF4 – EDF4 | 0.81 | 0.99 |
| CF5 – EDF5 | 1.07 | 0.96 |

Supplementary table 11: Summary of the multi-level generalized model used to compare the observed diversity of the fugal community of the different groups.

| Comparison between groups | F value | p-value |
| --- | --- | --- |
| CF2 – CF5 | 4.04 | 0.04 |
| CF2 – CF3 | 1.1 | 0.37 |
| CF3 – CF4 | 0.29 | 0.95 |
| CF4 – CF5 | 1.53 | 0.19 |
| EDF2 – EDF5 | 0.48 | 0.84 |
| EDF2 – EDF3 | 0.56 | 0.8 |
| EDF3 – EDF4 | 0.63 | 0.67 |
| EDF4 – EDF5 | 0.76 | 0.62 |
| CF2 – EDF2 | 1.45 | 0.23 |
| CF3 – EDF3 | 1.73 | 0.06 |
| CF4 – EDF4 | 3.11 | < 0.01 |
| CF5 – EDF5 | 3.94 | 0.02 |

*Supplementary table 12:* Summary of the multi-level generalized model used to compare the beta diversity of the fugal community of the different groups.
